# Complexity in the regulation of Dicer expression: Dicer variant proteins are differentially expressed in epithelial and mesenchymal breast cancer cells and decreased during EMT

**DOI:** 10.1038/sj.bjc.6606022

**Published:** 2010-11-30

**Authors:** G W Hinkal, G Grelier, A Puisieux, C Moyret-Lalle

**Affiliations:** 1 Centre Léon Bérard, Lyon, F-69008, France; 2 Université de Lyon, Lyon, F-69003, France; 3 Inserm, U590, Lyon, F-69008, France; 4 Université Lyon 1, ISPB, Lyon, F-69033, France; 5 Université Lyon 1, IUF, Lyon, F-69003, France

**Sir,**

In their recent article, Martello *et al* (2010) have shown that the miR-103/107 microRNA family downregulates global miRNA biogenesis by targeting the expression of the miRNA-processing endonuclease Dicer. They further show that Dicer downregulation leads to an epithelial-mesenchymal transition (EMT) and metastasis. Although Martello *et al* (2010) successfully elucidated a mechanism of Dicer downregulation, in this correspondence we extend our previous work (Grelier *et al*, 2009), and attempt to bring the field further by addressing several new questions and showing new results.

The novelty of the association of decreased Dicer expression with EMT and mesenchymal phenotype is questionable considering our previous published work. Using 21 breast cancer cell lines, two cellular models of human and mouse mammary cancer progression, and human cohort data, we had shown previously a significant downregulation of Dicer expression when cells exhibit mesenchymal phenotypes (Grelier *et al*, 2009). Using RT-qPCR and western blotting, we assessed Dicer expression in two distinct bone metastasis subpopulations derived from MDA-MB-231 breast cancer cells and found it to be significantly decreased in the two independent clones. Also, our examination of a cohort of breast cancer patients revealed significantly lower 8-year metastasis-free survival from primary tumours exhibiting lower Dicer cDNA levels.

As levels of the full-length protein were decreased with mesenchymal phenotypes, we wondered about the deregulated miRNAs targeting the Dicer 3′UTR during EMT, as it was already published that this region contains multiple different miRNA target sites. Dicer mRNA presents several let-7 target sequences, both in the coding region and in its 3′UTR (Figure 1A), differentially affecting its protein expression (Forman *et al*, 2008). Such a negative feedback regulation was shown on *Dicer-Like1* expression in Arabidopsis (Xie *et al*, 2003). Also, Asirvatham *et al* (2008) had previously shown, using multiple computational prediction tools, that the Dicer 3′UTR contains several miR-103/107 target sites. Using TargetScan, we have now found 27 different miRNA target sites in the 3′UTR, with five corresponding to the miR-103/107 (Figure 1A).

In addition to regulation via its long 3′UTR, mammalian Dicer mRNA appears as multiple spliced isoforms exhibiting 5′ and 3′ end truncations. We previously described 14 putative variants in human Dicer mRNA (Grelier *et al*, 2009). Notably, three variants encode the full-length protein, but only variants a and b exhibit the long 3′UTR sequence, while variant c has a very short 3′UTR lacking all predicted miRNA target sites (Figure 1A). We also identified two truncated forms, variants d and e (Figure 1A), whose expression vary greatly among different breast cancer cell lines (Figure 1B and data not shown). These two forms, respectively, encode a protein of 113 kDa and a protein of 92.7 kDa, which retain the ribonuclease III functional domains (Figure 1A). Variant d exhibits a 3′UTR sequence composed of a partial intron 23 and variant e has a very short 3′UTR sequence (30 bases), neither with any described miRNA binding sites. Here, we have shown that the presence of these spliced forms correlated with breast cancer staging subtypes and epithelial/mesenchymal phenotype (Figure 1B). Indeed, in almost all cell lines we examined that exhibit a complete or partial mesenchymal phenotype, these truncated isoforms were not detectable by western blot. Conversely, and without exception, epithelial cells expressed readily detectable levels of the two variants (Figure 1B). The absence of variants d and e correlated with basal cancer subtype cells; all luminal A subtype cells showed expression of the two alternative proteins; luminal B subtype cells showed higher expression of variant e (Figure 1B and data not shown). Furthermore, we have found decreased expression of variants d and e during EMT using immortalised human epithelial mammary cells transfected by RAS (Figure 1C). These data imply an integral role for internal site miRNA regulation of Dicer isoforms, but the physiological relevance of these data remains to be clarified.

In contrast to our results, Martello *et al* (2010) were not able to find Dicer mRNA expression associated with metastatic relapse in breast cancers, while they have shown an association between high levels of miR-103/107 and poor prognosis. In our study, Dicer mRNA expression was investigated by RT-qPCR using primers that specifically amplify the three full-length variants and not by transcriptomic microarrays that do not discriminate between these diverse forms of Dicer. Because variant c is insensitive to miRNA that targets 3′UTR, this could explain the discrepancy between 3′UTR-targeted Dicer downregulation and the high levels detected of mature miR-103/107. However, variant c can still be targeted by let-7 miRNAs in its coding sequence (Figure 1A) and, as implied by computational evidence, may lead to mRNA degradation (Forman *et al*, 2008). As two out of the three coding region let-7 binding sites are present on the Dicer variants a, b, c, d and e, this route cannot be ignored.

Many questions remain unaddressed with regard to Dicer mRNA regulation. What is the relevance of the Dicer and miR-103/107 expression relationship *in vivo* where Dicer exists as multiple mRNA isoforms and can be targeted by multiple miRNAs? Is there a competition between the different miRNAs in binding their targets as some miRNA consensus sequences overlap? Is there a competition between miRNA binding and the HuR RNA binding protein also present in 3′UTR of Dicer?

In summary, both our results (Grelier *et al*, 2009) and those of Martello *et al* (2010) show that Dicer downregulation is associated with EMT, a process that appears central to metastasis. Here we provide new results highlighting the complexity of Dicer expression regulation in breast cancer progression.

## Figures and Tables

**Figure 1:**
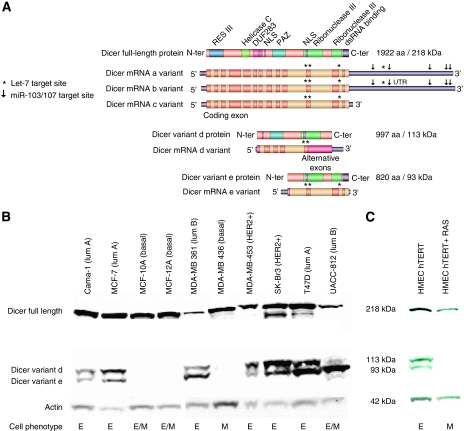
(**A**) Schematic representation of five Dicer mRNA variants and the corresponding proteins. Using AceView (http://www.AceView.org), transcription from the *Dicer* gene putatively produces 14 mRNAs, 11 alternatively spliced variants and 3 full-length forms. The cartoon shows the three full-length mRNA variants (a, b and c) and the corresponding full-length protein (218 kDa) and the two alternatively spliced variants (d and e) and their corresponding proteins (respectively, 113 and 93 kDa). For the mRNA, alternating colours indicate the different exons and grey bars represent 5′ and 3′UTRs. Domain structures are represented on proteins: green areas represent the ribonuclease domains, grey areas represent the dsRNA binding domain, and light green areas represent the helicase C domain. ↓ indicates miR-103/107 target sites in the 3′UTR region, ^*^indicates let-7 target sites in the coding and 3′UTR regions. 3′UTR variant a is 4269 bases long, 3′UTR variant b is 4270 bases long, 3′UTR variant c is 175 bases long and contains no miRNA target sites and ^**^indicates two let-7 sites. The 3′UTR of variant d is 431 bases long (corresponding to the partial intron 23 sequence) and the 3′UTR of variant e is 30 bases long (with no miRNA target sites). (**B**) Expression of full-length Dicer protein and d+e isoforms in breast cancer cell lines. Western blot analysis of full-length and truncated variant isoforms of Dicer levels in 10 breast cancer cell lines. Actin was used as a loading control. (**C**) Expression of full-length Dicer and d and e isoform proteins during EMT. Western blot analysis of Dicer expression in HMEC+hTERT (immortalised human mammary epithelial cells stably expressing hTERT) cell line and in HMEC+hTERT+RAS (immortalised HMEC+hTERT transfected with RAS oncogene) cells. Actin was used as a loading control. (E) indicates epithelial phenotype; (M) indicates mesenchymal phenotype; (E/M) indicates a mixed phenotype.
